# Expansion and activation of distinct central memory T lymphocyte subsets in complex regional pain syndrome

**DOI:** 10.1186/s12974-019-1449-9

**Published:** 2019-03-18

**Authors:** Marc A. Russo, Nathan T. Fiore, Caryn van Vreden, Dominic Bailey, Danielle M. Santarelli, Helen M. McGuire, Barbara Fazekas de St Groth, Paul J. Austin

**Affiliations:** 1 Hunter Pain Clinic, 91 Chatham Street, Broadmeadow, NSW 2292 Australia; 2 Genesis Research Services, 220 Denison St, Broadmeadow, NSW 2292 Australia; 3 Discipline of Anatomy & Histology, School of Medical Sciences, Faculty of Medicine and Health, The University of Sydney, Room E513, Anderson Stuart Building, Sydney, NSW 2006 Australia; 4 Ramaciotti Centre for Human Systems Biology, Charles Perkins Centre, The University of Sydney, Sydney, NSW 2006 Australia; 5 Sydney Cytometry, Centenary Institute and the Charles Perkins Centre, John Hopkins Drive, Camperdown, NSW 2050 Australia; 6 Discipline of Pathology, School of Medical Sciences, Faculty of Medicine and Health, The University of Sydney, Sydney, NSW 2006 Australia

**Keywords:** Complex regional pain syndrome, Central memory T lymphocytes, Myeloid dendritic cells, Mass cytometry, NF*k*B

## Abstract

**Background:**

Complex regional pain syndrome (CRPS) is a debilitating condition where trauma to a limb results in devastating persistent pain that is disproportionate to the initial injury. The pathophysiology of CRPS remains unknown; however, accumulating evidence suggests it is an immunoneurological disorder, especially in light of evidence of auto-antibodies in ~ 30% of patients. Despite this, a systematic assessment of all circulating leukocyte populations in CRPS has never been performed.

**Methods:**

We characterised 14 participants as meeting the Budapest clinical criteria for CRPS and assessed their pain ratings and psychological state using a series of questionnaires. Next, we performed immunophenotyping on blood samples from the 14 CRPS participants as well as 14 healthy pain-free controls using mass cytometry. Using a panel of 38 phenotypic and activation markers, we characterised the numbers and intracellular activation status of all major leukocyte populations using manual gating strategies and unsupervised cluster analysis.

**Results:**

We have shown expansion and activation of several distinct populations of central memory T lymphocytes in CRPS. The number of central memory CD8^+^ T cells was increased 2.15-fold; furthermore, this cell group had increased phosphorylation of NF*k*B and STAT1 compared to controls. Regarding central memory CD4^+^ T lymphocytes, the number of Th1 and Treg cells was increased 4.98-fold and 2.18-fold respectively, with increased phosphorylation of NF*k*B in both populations. We also found decreased numbers of CD1c^+^ myeloid dendritic cells, although with increased p38 phosphorylation. These changes could indicate dendritic cell tissue trafficking, as well as their involvement in lymphocyte activation.

**Conclusions:**

These findings represent the first mass cytometry immunophenotyping study in any chronic pain state and provide preliminary evidence of an antigen-mediated T lymphocyte response in CRPS. In particular, the presence of increased numbers of long-lived central memory CD4^+^ and CD8^+^ T lymphocytes with increased activation of pro-inflammatory signalling pathways may indicate ongoing inflammation and cellular damage in CRPS.

## Background

Complex regional pain syndrome (CRPS) is a disease that has perplexed clinicians and basic scientists alike, not only for the refractory nature of the condition but also for its protean nature. It can manifest in any given individual, although it is more common in females, and many of its features are difficult to explain on a pathological level. This has led to some clinicians even declaring that the condition is psychosomatic in origin [1, 2]. CRPS clearly presents as a neuropathic pain condition but is mostly singularly unresponsive to oral anti-neuropathic agents (e.g. Gabapentin, Pregabalin). It has classical features that mostly occur after trauma or surgery to a distal limb, and that start in the periphery and ascend to the groin in lower limb cases and to the axilla in upper limb cases, becoming geographically demarcated at these levels. Traditional subtyping of CRPS depends on whether disease onset is associated with a clinically confirmed direct peripheral nerve injury (CRPS type II—previously ‘causalgia’) or without a confirmed nerve injury (CRPS type I—previously ‘reflex sympathetic dystrophy’); however, research indicates that CRPS-I also involves nerve trauma [3–5]. Regardless of subtype, the pain is disproportionate to the initiating event and is persistent. It is associated with some degree of swelling of the distal limb, colour change, temperature change, sweating change and motor impairment (weakness, tremor, dystonic posture). A ‘CRPS-not otherwise specified’ label may be applied to those few patients in whom some of these symptoms resolve over time but significant pain remains [6].

Whilst CRPS was initially considered a neurological phenomenon, there is accumulating evidence that it is an immunoneurological disorder [7–9]. Early evidence for this can be found as far back as the 1950s in the response to oral corticosteroid administration in early presentations of the condition (< 6 months) [10]. In most cases, acute CRPS resembles peripheral inflammation. The inflammatory response is excessive in CRPS, and this can have pathological effects on the nervous system and lead to perpetuating inflammation and chronic pain [11–13]. Multiple individual findings of immune system involvement have been documented. This has included increased expression of pro-inflammatory cytokines in the skin, blood and cerebrospinal fluid of CRPS patients [14–19]. There is evidence for local neurogenic inflammation, with elevation of neuropeptides, particularly CGRP and Substance P that mediate oedema and inflammation and may lead to an immune mediator cascade [20–22]. Auto-antibody production has also been implicated, especially involving the muscarinic-2 acetylcholine (M2) receptor, and the *α*_1a_ and *β*_2_ adrenoreceptors [8, 23–26], whilst elevated plasma levels of soluble IL-2 suggest a possible T lymphocyte-mediated response [27]. A recent review has summarised the auto-inflammatory and auto-immune contributions to CRPS (see [28]).

Flow cytometry has been used to investigate the immune function in CRPS patients with mixed results [29–32]. These studies analysed a small subset of immune cell types and did not investigate intracellular signalling. An alternative approach to assess immune function is via mass cytometry, which uses metal isotopes instead of fluorophores as reporters, increasing the multiplexing capacity and allowing a large array of immune cells and intracellular activation markers to be examined concurrently. Mass cytometry is a powerful tool that is allowing for new insights into cell biology and systems function to be gained [33–38]. Therefore, the current study assessed peripheral blood mononuclear cells (PBMCs) and their intracellular activation status using mass cytometry and systematic unsupervised analysis approaches in chronic (> 6 months) CRPS patients and control subjects.

## Methods

### Participants

Participants were recruited by Genesis Research Services (Broadmeadow, NSW, Australia) between September 2017 and April 2018, and signed informed consent. The study included both male and female participants aged 19–70 years old, who met the Budapest Criteria for clinical CRPS diagnosis [39] (*n* = 14, Table 1) or were age-matched pain-free controls (*n* = 14). We chose to investigate the long-standing immune changes in cases of CRPS (> 6 months), rather than acute changes associated with the initial trauma and healing (< 6 months). Exclusion criteria were less than 18 years of age; CRPS symptom onset less than 6 months (CRPS group); any acute pain (control group); any other neurological, psychiatric or pain conditions that could confound the study endpoints; and pregnancy. On the day of blood collection, participants had ceased taking immune modulating medications (e.g. NSAIDs, steroids and opioids) for at least 7 days. The 7-day drug wash-out could still leave residual effects on immune cells, especially lymphocytes; however, it was the longest wash-out practicable.

**Table 1:** A table showing the Budapest Criteria for clinical CRPS diagnosis and which symptoms and signs were present in our cohort. All four criteria must be met in order to make a clinical diagnosis of CRPS according to the Budapest Criteria [39]. On this basis, 14 participants were clinically diagnosed with CRPS in our study

	Criteria	Number of participants fulfilling each category			
		Sensory	Vasomotor	Sudomotor	Motor/trophic
1	Continuing pain, disproportionate to any inciting event	14			
2	Symptoms: Must report at least one symptom in three of the four categories shown to the right	14 (Hyperesthesia; allodynia)	9 (Temperature asymmetry; changes in skin colour; skin colour asymmetry)	14 (Edema; sweating changes; sweating asymmetry)	12 (Decreased range of motion; motor dysfunction; trophic changes (hair, nails, skin))
3	Signs: At the time of evaluation, must have at least one sign in two or more of the categories shown to the right	11 (Hyperalgesia (pinprick); allodynia (light touch or temperature); deep somatic pressure; joint movement)	11 (Skin temperature asymmetry (> 1 °C); changes in skin colour; skin colour asymmetry)	8 (Edema; sweating changes; sweating asymmetry)	7 Decreased range of motion; motor dysfunction (weakness, tremor, dystonia); trophic changes (hair, nails, skin))
4	No other diagnosis can better explain the patient’s signs and symptoms	14			

### Pain and psychological profiling

All participants rated pain from 0 to 100 on a visual analogue scale (VAS) and completed 5 questionnaires to assess pain and psychological variables: Short form McGill pain questionnaire (SF-MPQ-2), short form depression, anxiety and stress Scale (DASS21), Pain self-efficacy questionnaire, (PSEQ), Tampa scale for kinesiophobia (TSK) and Pain catastrophizing scale (PCS).

### Blood collection protocol

Blood was taken by venepuncture from the unaffected limb antecubital fossa by a trained phlebotomist, and collected into a 5 ml ethylenediaminetetraacetic acid (EDTA) tube and inverted immediately. Blood was immediately transferred to ten 1.5 ml tubes, 0 .5 ml in each, that contained 0 .7 ml of proteomic stabliser buffer (Smart Tube Inc., CA, USA), and mixed by inversion. Samples were then incubated in proteomic stabliser buffer at room temperture for 10 min, before being transferred to a − 80 °C freezer.

### Mass cytometry staining

Blood samples in 1.5 ml tubes were taken out of − 80 °C and transferred to the fridge to thaw samples for 30–45 min. Once thawed, 1 ml of Thaw-Lyse buffer 1 (Smart Tube Inc) was added to each tube and thoroughly mixed. Two 1.5 ml tubes from the same participant (total of 1 ml original blood volume) were combined in a 15 ml tube, topped up to 10 ml with Thaw-Lyse buffer 1, and left at room temperature for 10 min. Samples were spun at 600×*g* at 4 °C, the supernatant was removed and then the cells lysed a second time with 10 ml Thaw-Lyse buffer 1. Samples were then lysed with 10 ml of Lyse Buffer 2 (Smart Tube Inc.). At this point, if the pellet was not completely white (i.e. incomplete red blood cell lysis), an extra lysis step with Lyse Buffer 2 (Smart Tube Inc) was undertaken. Cells were then suspended in fluorescence-activated cell sorting (FACS) buffer (PBS with 5% FCS, 5 mM EDTA and 0.2% sodium azide) and counted with a haemocytometer to calculate total leukocytes per ml of blood.

Next, 2.5 × 10^6^ cells were transferred to a 1.5 ml tube and incubated with 200 μl heparin (100 IU/ml) in FACS buffer for 20 min at room temperature. Heparin reduces non-specific eosinophil staining artefacts in mass cytometry [40]. Cells were then washed with FACS buffer, spun at 500×*g* for 5 min and the supernatant was removed before being incubated with surface antibodies on ice for 30 min (see Table 1 for antibody manufacturers and concentrations). Note, the concentrations for all antibodies were optimised for use with the proteomic stabliser buffer prior to staining the experimental samples. Cells were washed and incubated with fixation buffer (eBioscience) for 45 min on ice. Cells were then washed twice in permeabilization buffer (eBioscience), before being incubated with 200 μl heparin (100 IU/ml) in permeabilization buffer for 20 min at room temperature. Cells were washed and spun at 800×*g* for 7 min, and the supernatant was removed before incubation with intracellular antibodies for forkhead box P3 (FoxP3), T-box protein expressed in T lymphocytes (T-bet), Arg-1 and DAP12 for 30 min on ice (Table 2). Cells were then washed once with permeabilization buffer and then with FACS buffer, before incubation with 500 μl ice-cold methanol for 30 min on ice. Cells were then washed in FACS buffer before being incubated with 200 μl heparin (100 IU/ml) in FACS buffer for 20 min at room temperature. Cells were washed and then incubated with antibodies against intracellular signalling markers for 30 min on ice (Table 1). Cells were washed before incubation with an anti-PE antibody for 15 min on ice. This was necessary as we used a pPLC*γ*2-PE antibody instead of a direct metal conjugated pPLC*γ*2 antibody. Cells were washed before being incubated overnight in the fridge with 200 μl DNA intercalator (Cell ID, Fluidigm) diluted 1:4000 in paraformaldehyde (PFA).

**Table 2:** A table showing the mass cytometry panel used to immunophenotype the blood of CRPS and healthy control participants. The columns represent the rare-earth metal isotype used for conjugation, species reactivity, target antigen, clone, manufacturer and staining concentration. The antibody from Fluidigm was purchased metal labelled, and antibodies from all other manufacturers were purchased, metal-conjugated and validated in-house by the Ramaciotti Facility for Human Systems Biology

Metal isotype	Reactivity	Antigen	Clone	Manufacturer	Staining conc. (μg/ml)	Staining step buffer
^110^Pd	Mouse, Human	CD45	30-F11	BD	6	FACS
^115^In	Human	CD8	RPA-T8	Biolegend	4	FACS
^141^Pr	Human	CD235ab	HIR2	Biolegend	1.6	FACS
^142^Nd	Human	CD19	HIB19	BD	1	FACS
^143^Nd	Human	CD56	REA196	Miltenyi Biotec	0.5	FACS
^144^Nd	Human	TCR *γδ*	B1	BD	2	FACS
^145^Nd	Human	CD4	RPA-T4	BD	0.5	FACS
^146^Nd	Human	DAP12	406288	R&D systems	1	Perm. buffer
^147^Sm	Human, Mouse	TREM-2	237920	R&D systems	8	FACS
^148^Nd	Human	CD16	3G8	BD	1.5	FACS
^149^Sm	Human	CD25	2A3	BD	1	FACS
^150^Nd	Mouse, Human	pSTAT5 [pY694]	47	BD	1.5	Methanol
^151^Eu	Human	CD123 (IL-3R)	6H6	Biolegend	1	FACS
^152^Gd	Human	CD66b	80H3	Fluidigm	3 (μl/ml)	FACS
^153^Eu	Mouse, Human	pSTAT1 [pY694]	14/P-Stat1	BD	2	Methanol
^154^Gd	Mouse, Human	pAKT [T308]	J1.233.371	BD	4	Methanol
^155^Gd	Human	CD354 (TREM-1)	TREM-37	Biolegend	2	FACS
^156^Gd	Human	pp38 MAPK [T180/Y182]	30/p38 MAPK	BD	4	Methanol
^158^Gd	Human, Mouse	pSTAT3 [Y705]	4/P-STAT3	BD	20	Methanol
^159^Tb	Human	CD197 (CCR7)	G043H7	Biolegend	8	FACS
^160^Gd	Human	CD14	M5E2	Biolegend	6	FACS
^161^Dy	Human	CD141 (BDCA3)	AD5-14H12	Miltenyi Biotec	8	FACS
^162^Er	Human	Foxp3	PCH101	eBioscience	3	Perm. buffer
^163^Dy	Human	CD1c	L161	Biolegend	1	FACS
^164^Er	Human	CD45RO	UCHL1	Biolegend	6	FACS
^165^Ho	Human	CD61 (Integrin *β* 3)	VI-PL2	Biolegend	1	FACS
^166^Er	Human	pp65 (NF*κ*B) [S529]	K10-895.12.50	BD	4	Methanol
^167^Er	Human	CD11c	Bu15	Biolegend	1	FACS
^168^Er	Human	pERK 1/2 [Y202/204]	4B11B69	Biolegend	2	Methanol
^169^Tm	Human	CD45RA	HI100	Biolegend	2	FACS
^170^Er	Human	CD3	UCHT1	BD	2	FACS
^171^Yb	Human	Arginase-1	14D2C43	Biolegend	4	Perm. buffer
^172^Yb	Human	CD130	AM64	BD	8	FACS
^173^Yb	Human, Mouse	pMAPKAPK-2 [T334]	P24-694	BD	4	Methanol
^174^Yb	Human	HLA-DR	G46-6	BD	2	FACS
^175^Lu	PE	PE	PE001	Biolegend	4	FACS
^176^Lu	Human	CD127	A019D5	Biolegend	2	FACS
^209^Bi	Human, Mouse	T-bet	4B10	BD	1	Perm. buffer
n/a	Human	pPLC *γ* 2 [Y759]-PE	K86-689.37	BD	200 (μl/ml)	Methanol

### Running mass cytometry

On the day of sample acquisition, the cells were diluted in 1 ml FACS buffer and washed. The cells were then washed twice in MilliQ water, and then re-suspended at 1 × 10^6^/ml in 1:10 EQ calibration beads (Fluidigm). A minimum number of 500,000 events were then acquired on a mass cytometer (Helios CyTOF, Fluidigm) using CyTOF software (Fluidigm). Following acquisition, all flow cytometry standard (.fcs) files were normalised to bead signal levels using CyTOF software (see [41] for more details).

### Manual gating

**Fig. 1:**
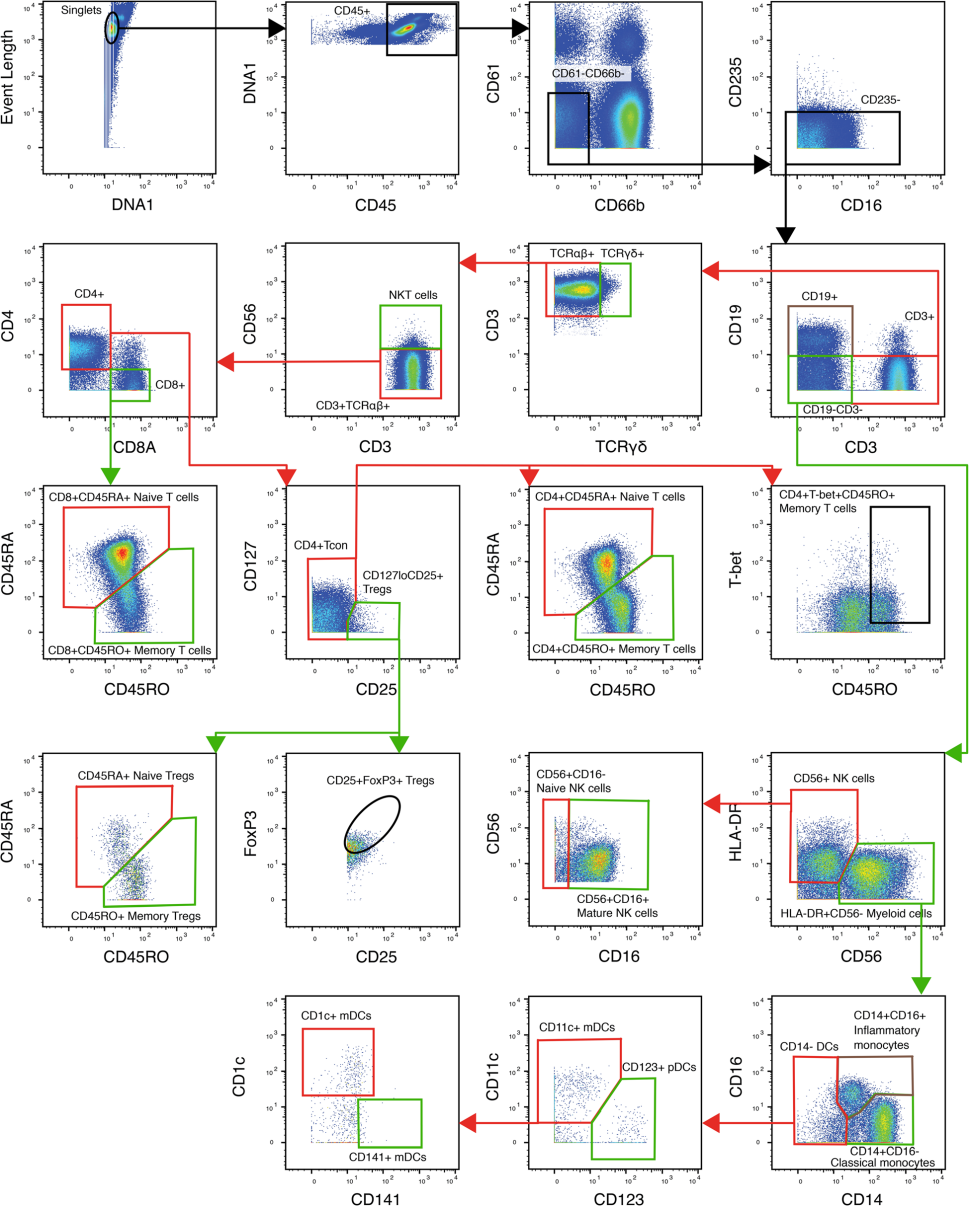
A flow diagram representing the manual gating strategy used to isolate major immune cell populations from mass cytometry output. Firstly, quality control gates were performed to remove normalisation beads, isolate singlets and gate CD45^+^ leukocytes. Next, mononuclear leukocytes (CD66^−^CD61b^−^CD235^−^) were isolated by gating out platelets, granulocytes and erythrocytes. Major lymphocyte and myeloid populations were then isolated using standard phenotypic markers. For example, CD19^+^ for B lymphocytes, CD3^+^ for T lymphocytes, CD56^+^ for NK cells and HLA-DR^+^ for myeloid cells. Finally, major cell populations were gated further into known subsets

Using FlowJo 10 (OR, USA), normalisation beads were gated out, and then singlets were gated on event length and DNA1. Leukocytes were gated using CD45^+^ and DNA1. From the CD45^+^ population, CD66b^−^CD61b^−^ mononuclear leukocytes were gated, and red blood cell contamination was eliminated by further gating a CD235^−^ population. Major immune cell populations were gated using a manual gating strategy (Fig. 1). All absolute cell numbers of mononuclear cells per ml of blood were back-calculated from the total leukocyte counts made following red blood cell lysis.

### SPADE and FlowSOM

For all data sets, pre-gating to exclude doublets, debris, platelets and red blood cells was performed (top row of Fig. 1). Data was also pre-processed via application of an arcsinh conversion with a standard co-factor of 5. To visualise global changes in intracellular markers in CRPS participants, spanning tree progression of density-normalised events (SPADE) clustering algorithm were run on remaining single mononuclear leukocytes; no additional pre-gating was performed [42]. In brief, the SPADE tree was built with a maximum downsampling population of 50,000 cells and a *k* means of 100. The locations of clusters representing major leukocyte populations within the SPADE trees were first identified by visualising SPADE trees for all major phenotypic markers, e.g. CD3 for T lymphocytes, CD19 for B lymphocytes, CD14 for classical monocytes. Combinations of phenotypic markers could be used to identify smaller subsets within the SPADE trees, e.g. CD14 and CD16 for inflammatory monocytes. Once these leukocyte populations were identified, SPADE trees displaying intracellular markers could be interpreted.

To analyse the cellular identity and marker expression characteristics of similar cells within a node, phenotypical clustering analysis via a self-organising map (FlowSOM) was performed on single live cells that were pre-gated on CD3^+^ or HLA-DR^+^. FlowSOM clustering was conducted via the cytofkit-package (https://github.com/JinmiaoChenLab/cytofkit), which integrates the FlowSOM algorithm from the FlowSOM package [43]. In brief, each set of pre-gated cells were initially organised into 40 clusters and then phenotypically similar nodes were grouped into meta-clusters to allow identification of the cellular identity of each node.

### Statistical analysis

Fisher’s exact test was used to compare the proportion of male and female participants between CRPS and control groups (Prism 7, GraphPad Software Inc.). All other data were first analysed for normality using the D’Agostino-Pearson normality test, and then an unpaired Student’s *T* test (for normally distributed data), or a Mann-Whitney *U* test (for non-normally distributed data) were used to test for statistically significant differences between control and CRPS groups (Prism 7). For families of analyses, the family-wise error rate was corrected using the Benjamini-Hochberg procedure, at a false discovery rate of *q* < 0.1. This is the most appropriate and stringent methodology to correct for multiple comparisons and has been used by similar studies [44, 45]. A ‘family of analyses’ was considered to be where a comparable biological measure was made multiple times on the same biological tissue (i.e. the expression of multiple intracellular signalling molecules within a cell population). The data from individual CRPS participants were also analysed using linear regression to determine significant relationships between changes in cell numbers, intracellular signalling and pain or psychological variables.

## Results

### Participants and clinical findings

All participants in the CRPS group met the Budapest diagnostic criteria for CRPS [39] and were in the chronic phase of disease, having symptoms for at least 6 months. Symptom onset in all 14 participants in the CRPS group occurred following trauma; 7 suffered fractures, 4 had soft tissue injuries and 3 had undergone surgery, and there was an equal proportion of participants with upper and lower limbs affected. CRPS is more common in females than males at a ratio of 3:1, and although the proportion of females in our CRPS group was greater than in the healthy control group, this was not statistically significant (*P* = 0.13). Severe pain was confirmed in the CRPS group, with a mean pain rating of 69/100 on a visual analogue scale (VAS) and a mean score of 114/220 on the Short-form McGill Pain Questionnaire (SF-MPQ2), both significantly higher than the control group (both *U* = 0, *P* < 0.001, Table 3). Psychological state was examined using the Short-form Depression, Anxiety and Stress scale (DASS21). According to the DASS21 severity ratings, the CRPS group had mild depression (*U* = 34.5, *P* < 0.01), moderate anxiety (*U* = 22.5, *P* < 0.001) and mild stress (*U* = 1, *P* < 0.001), all increased compared to the control group, which had normal scores on all ratings. Kinesiophobia, pain catastrophising and reduced pain self-efficacy were also found in the CRPS group, which had higher scores on the Tampa Scale for Kinesiophobia (TSK) and Pain Catastrophising Scale (PCS), and lower scores on the Pain Self-efficacy Questionnaire (PSEQ) compared to the control group (*U* = 14.5, *U* = 12.5 and *U* = 0 respectively, all *P* < 0.001).

**Table 3:** CRPS participants reported higher scores for pain, anxiety depression, stress, pain catastrophising and kinesiophobia, as well as reduced pain self-efficacy compared to healthy controls. A table showing participant information, pain and psychological measures in CRPS participants and healthy controls. Visual analogue scale (VAS), Short-form Depression, Anxiety and Stress scale (DASS21), Short-form McGill Pain Questionnaire (SF-MPQ-2), Tampa Scale for Kinesiophobia (TSK) and Pain Catastrophising Scale (PCS), Pain Self-efficacy Questionnaire (PSEQ). *N* = 14 CRPS and *N* = 14 controls. All data are presented as group means (± S.E.M.). ** represents *P* < 0.01, *** represents *P* < 0.001 in in a Mann-Whitney *U* test

		Control ± S.E.M.	CRPS ± S.E.M.
Sex (F/M)		5/9	10/4
Age (years)		39.21 ± 3.3	46.14 ± 4.2
CRPS type (I/II)			14/0
Time since onset (years)			4.13 ± 1.6
Limb affected (upper/lower)			7/7
CRPS trigger (fracture/soft tissue injury/surgery)			7/4/3
Pain score (VAS 0–100)		7.6 ± 1.1	69.36 ± 3.7***
DASS 21 (0–42)	Depression	2.8 ± 1.2	11.14 ± 2.8**
	Anxiety	3.0 ± 0.8	14.00 ± 2.0***
	Stress	5.8 ± 1.8	17.58 ± 2.0***
SF-MPQ-2 (0–220)		4.8 ± 2.0	113.71 ± 11.0***
PCS (0–52)		3.2 ± 1.6	21.57 ± 2.8***
PSEQ (60–0)		57.4 ± 1.1	24.07 ± 2.9***
TSK (0–68)		29.1 ± 1.6	39.79 ± 1.3***

### Manual gating results

A manual gating strategy was used to quantify changes in major leukocyte populations that would undergo subsequent validation using unsupervised cluster analyses. In total, we quantified 22 distinct immune subsets in the peripheral blood of CRPS patients, a magnitude more than previous studies. This approach identified higher numbers of total CD3^+^ (excluding TCR*γδ* ^+^) T lymphocytes in the CRPS group (*t*_26_ = 2.139, *P* < 0.05, Table 4). Further analysis of major T lymphocyte subsets revealed significantly higher numbers of CD4^+^CD127^lo^CD25^+^FoxP3^+^ Tregs compared to control (*t*_26_ = 2.286, *p* < 0.05). CD4^+^CD45RO^+^T-bet^+^ Th1 cells were twice as numerous in the CRPS group; however, this dataset was not normally distributed and did not reach statistical significance (*U* = 65, *P* = 0.14). There were no significant differences in classical and inflammatory monocytes or B, NK and NKT cell numbers. Surprisingly, there were reduced numbers of CD11c^+^CD1c^+^CD141^−^ myeloid dendritic cells (mDCs) in the CRPS group (*U* = 44.5, *P* < 0.05).

**Table 4:** Significant increases in major T lymphocyte populations in the blood of CRPS participants. A table showing the numbers of major immune cell populations in the blood of CRPS and healthy controls based on manual gating analysis of mass cytometry output. *N* = 14 CRPS and *N* = 14 controls. All data are presented as group means (± S.E.M.). * represents *P* < 0.05 in unpaired Student’s *T* test, ^#^ represents *P* < 0.05 in Mann-Whitney *U* test

Cell population	Control (cells/ml)	± S.E.M.	CRPS (cells/ml)	± S.E.M.
CD3^+^TCR*γδ*^−^ T cells	446,868	34,128	657,776*	92,487
CD3^+^TCR*γδ*^−^CD4^+^ T cells	263,243	20,573	396,510	63,516
CD4^+^CD45RA^+^ Naive T cells	103,023	13,699	154,529	26,565
CD4^+^CD45RO^+^ Memory T cells	144,787	11,592	208,773	39,029
CD4^+^CD45RO^+^T-bet^+^ Memory Th1 cells	51,318	6575	101,242	23,548
CD4^+^CD127^lo^CD25^+^ Tregs	14,782	2127	32,343	9936
CD4^+^CD127^lo^CD25^+^CD45RA^+^ Naive Tregs	4255	909	8190	2653
CD4^+^CD127^lo^CD25^+^CD45RO^+^ Memory Tregs	10,421	1365	23,934	7461
CD4^+^CD127^lo^CD25^+^FoxP3^+^ Tregs	5573	485	11,925*	2736
CD3^+^TCR*αβ* ^+^ CD8^+^ T cells	183,550	18,908	261,338	40,889
CD8^+^CD45RA^+^ Naive T cells	108,545	16,263	142,125	23,135
CD8^+^CD45RO^+^ Memory T cells	74,962	7634	118,769	28,520
CD3^+^TCR*αβ* ^+^CD56^+^ NKT cells	10,092	1296	20,082	7371
CD3^+^TCR*γδ* ^+^ T cells	8384	1465	15,172	4273
CD19^+^ B cells	62,633	10,241	93,386	19,075
CD56^+^CD19^−^CD3^−^ NK cells	64,508	8369	103,364	19,520
HLA-DR^+^CD19^−^CD3^−^CD56^−^ Myeloid cells	199,230	28,780	260,322	33,995
HLA-DR^+^CD14^+^ Classical monocytes	158,330	24,822	207,083	29,342
HLA-DR^+^CD14^+^CD16^+^ Inflammatory monocytes	23,262	2984	28,884	3968
HLA-DR^+^CD14^−^CD11c^+^CD1c^+^CD141^−^ mDC	3101	433	1738^#^	344
HLA-DR^+^CD14^−^CD11c^+^CD141c^+^CD1c^−^ mDC	1030	196	883	119
HLA-DR^+^CD14^−^CD123^+^ pDCs	2624	322	2968	727

### Unsupervised cluster analysis of immune cells and intracellular activation

Unsupervised clustering analyses FlowSOM and SPADE were used to further probe all major adaptive and innate cell populations in CRPS, in particular to confirm significant changes identified with manual gating, but also to investigate more specific cell clusters, as well as their intracellular signalling activation. For the FlowSOM analysis, only clusters with statistically significant differences in cell numbers between control and CRPS are reported below.

#### CD8^+^ T lymphocytes

**Fig. 2:**
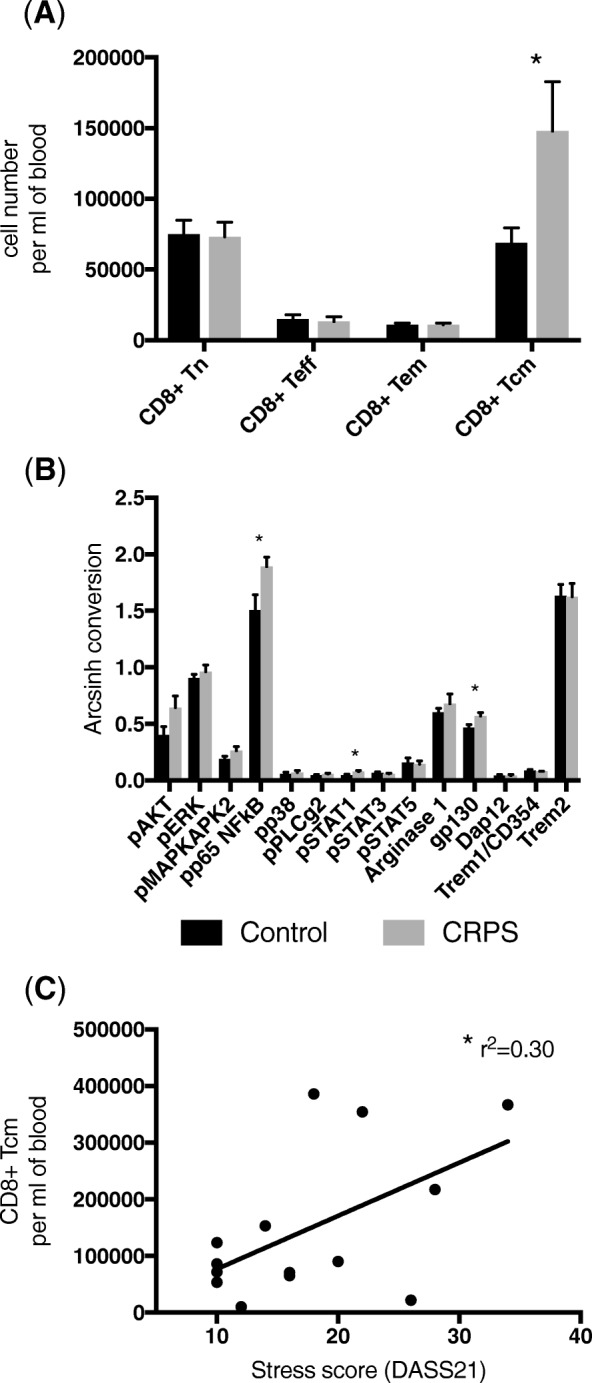
Increased numbers of central memory CD8^+^ T lymphocytes with increased pro-inflammatory activation are found in the blood of CRPS participants compared to healthy controls. **a** A bar graph showing the number of cells in FlowSoM clusters representing the four major CD8^+^ T lymphocyte populations; naive (Tn, CD3^+^CD8^+^CD45RA^+^CCR7^+^), effector (Teff, CD3^+^CD8^+^CD45RA^+^CCR7^−^), effector memory (Tem, CD3^+^CD8^+^CD45RO^+^CCR7^−^), central memory (Tcm, CD3^+^CD8^+^CD45RO^+^CCR7^+^). **b** Intracellular signalling and functional marker expression levels in the FlowSoM cluster representing central memory CD8^+^ T lymphocytes. All data are presented as group means (± S.E.M.). * represents *P* < 0.05 in an unpaired Student’s *T* Test. **c** Linear correlation between the number of central memory CD8^+^ T and stress scores on DASS21 in the CRPS group. *N* = 14 CRPS and N = 14 controls. * represents *P* < 0.05 in a linear regression analysis

FlowSOM analysis identified significantly higher numbers of central memory CD8^+^ T lymphocytes (CD3^+^CD8^+^CD45RO^+^CCR7^+^) in the CRPS group compared to controls (*t*_26_ = 2.163, *P* < 0.05), whereas the number of naïve, effector and effector memory CD8^+^ cells were stable across both groups (Fig. 2a). Moreover, the central memory CD8^+^ T lymphocytes cluster contained greater expression of phosphorylated p65 (subunit of NF*κ*B) and STAT1, as well as increased expression of gp130, compared to control (*t*_26_ = 2.384, *t*_26_ = 2.23 and *t*_26_ = 2.436 respectively, all *P* < 0.05, Fig. 2b). There was no correlation between pain scores (VAS or SF-MPQ2) and central memory CD8^+^ T lymphocytes within the CRPS group. However, the number of central memory CD8^+^ T lymphocytes in individuals in the CRPS group positively correlated with the stress scores on the DASS21 (*F*_1,12_ = 5.13, *r*^2^ = 0.30, *P* < 0.05, Fig. 2c). That is more central memory CD8^+^ T lymphocytes are associated with higher perceived stress levels in CRPS.

#### CD4^+^ T lymphocytes

**Fig. 3:**
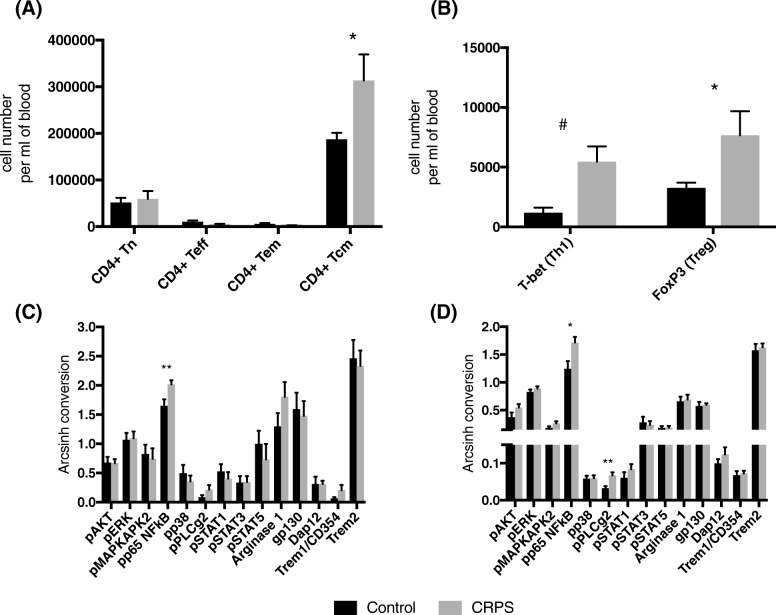
Increased numbers of central memory CD4^+^ T lymphocytes with increased pro-inflammatory activation are found in the blood of CRPS participants compared to healthy controls. **a** A bar graph showing the number of cells in FlowSoM clusters representing the four major CD4^+^ T lymphocyte populations; naive (Tn, CD3^+^CD4^+^CD45RA^+^CCR7^+^), effector (Teff, CD3^+^CD4^+^CD45RA^+^CCR7^−^), effector memory (Tem, CD3^+^CD4^+^CD45RO^+^CCR7^−^), central memory (Tcm, CD3^+^CD4^+^CD45RO^+^CCR7^+^). **b** A bar graph showing the number of cells in FlowSoM clusters representing central memory Th1 lymphocytes (CD3^+^CD4^+^CD45RO^+^CCR7^+^T-bet^+^) and Tregs (CD3^+^CD4^+^CD45RO^+^CCR7^+^CD127^lo^CD25^+^FoxP3^+^). Intracellular signalling and functional marker expression levels in FlowSoM clusters representing **c** Th1 lymphocytes and **d** Tregs. *N* = 14 CRPS and *N* = 14 controls. All data are presented as group means (± S.E.M.). * represents *P* < 0.05 and ** represents *P* < 0.01 in an unpaired Student’s *T* Test, and ^#^ represents *P* < 0.01 in a Mann-Whitney *U* Test

FlowSOM analysis identified higher numbers of central memory CD4^+^ T lymphocytes (CD3^+^CD4^+^CD45RO^+^CCR7^+^) compared to controls (*t*_26_ = 2.185, *P* < 0.05), whereas naïve, effector and effector memory were unchanged (Fig. 3a). Looking at more specific clusters, the number of central memory Th1 lymphocytes (CD3^+^CD4^+^CD45RO^+^CCR7^+^T-bet^+^) and central memory Tregs (CD3^+^CD4^+^CD45RO^+^CCR7^+^CD127^lo^CD25^+^FoxP3^+^) were greater in the CRPS group (*U* = 44, *P* < 0.05 and *t*_25_ = 2.208, *P* < 0.05 respectively; Fig. 3b). There was no correlation between pain scores (VAS or SF-MPQ2) and any CD4^+^ T lymphocyte populations within the CRPS group. Unexpectedly, the number of central memory Th1 lymphocytes in individuals in the CRPS group negatively correlates with anxiety scores on DASS21 (*F*_1,12_ = 7.7, *r*^2^ = 0.39, *P* < 0.05). Indicating that lower perceived anxiety levels occur in the patients with the greatest number of Th1 lymphocytes.

Several markers of intracellular expression were increased in the CRPS group. Expression of phosphorylated P65 in the central memory Th1 lymphocytes (*t*_26_ = 2.817, *P* < 0.01; Fig. 3c), and expression of phosphorylated p65 and 1-phosphatidylinositol-4,5-bisphosphate phosphodiesterase gamma-2 (PLC*γ*2) in central memory Tregs were increased compared to controls (*t*_25_ = 2.746, *P* < 0.05 and *t*_25_ = 3.467, *P* < 0.01 respectively; Fig. 3d). Further, expression of phospho-p65 in central memory Th1 lymphocytes was negatively correlated with PCS scores (*F*_1,12_ = 5.0, *r*^2^ = 0.29, *P* < 0.05), indicating reduced activation of nuclear factor kappa-light-chain-enhancer of activated B cells (NF*κ*B) in Th1 cells is associated with greatest pain catastrophising.

**Fig. 4:**
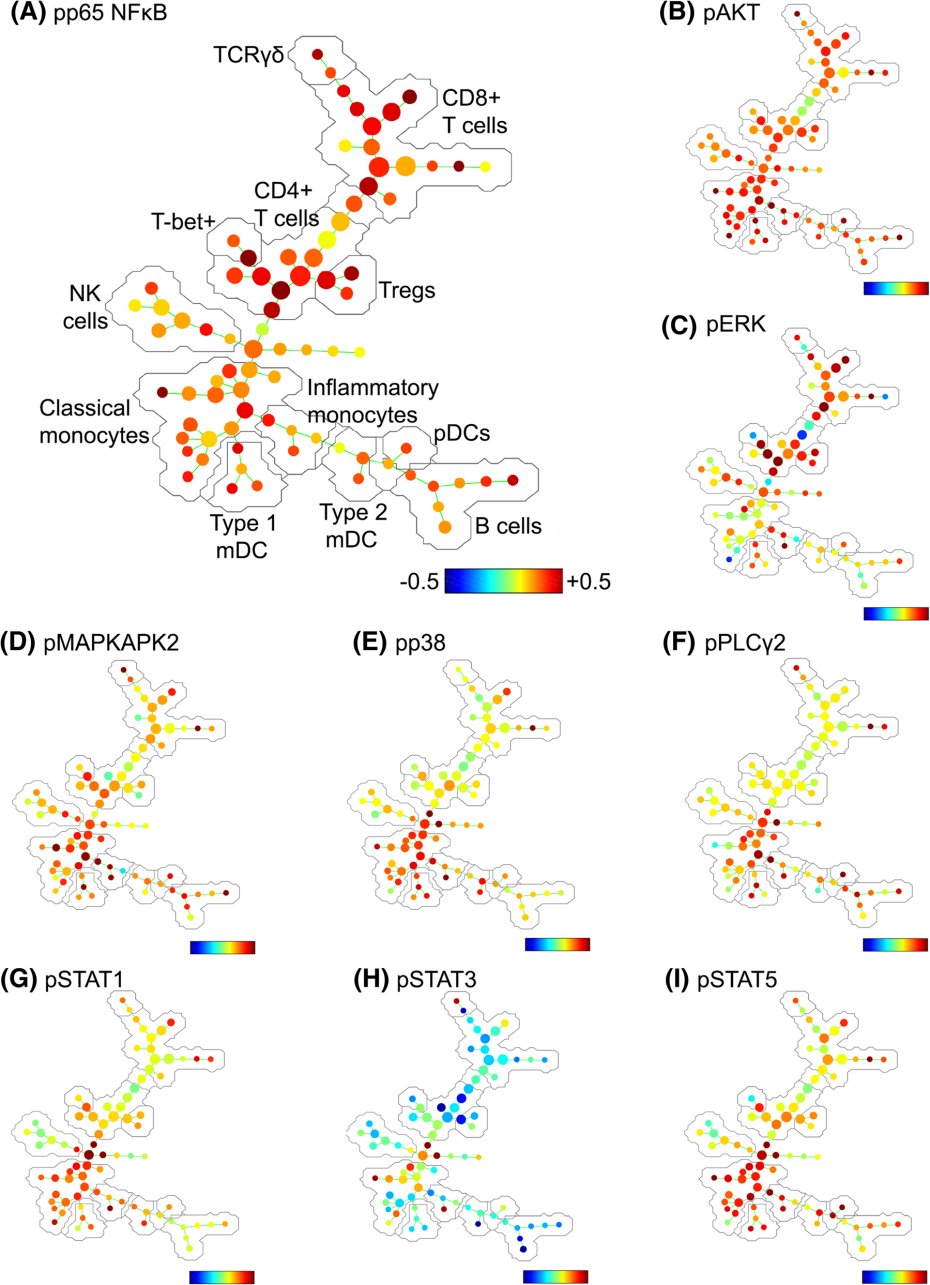
Spanning tree progression of density-normalised events (SPADE) trees showing evidence of pro-inflammatory activation in distinct lymphocyte and myeloid cell populations in the blood of CRPS participants relative to healthy controls. The SPADE algorithm was run on a downsampled population of single leukocytes from all CRPS and control participants. SPADE trees were generated showing the fold-change between CRPS and control groups in the expression of phosphorylated (activated), **a** p65 NF*κ*B, **b** AKT, **c** ERK, **d** MAPKAPK2, **e** p38, **f** PLC*γ*2, **g** STAT1, **h** STAT3 and **i** STAT5. The major cell populations labelled in (**a**) are representative of all SPADE trees. Note: colour scales vary for each marker

SPADE trees are a useful visualisation tool to overlay expression patterns of intracellular markers across major leukocyte populations. They are useful to highlight trends in expression patterns, although unlike FlowSOM clusters, they are not well suited for statistical comparison. In adaptive cell types, SPADE trees reveal a generalised increased in phospho-p65 expression in T lymphocytes (Fig. 4a), a moderate increase in phospho-ERK in some lymphocyte populations (Fig. 4c), and a generalised reduction in phospho-STAT3 expression, most apparent in CD4^+^ T lymphocytes (Fig. 4h).

#### Myeloid cells

FlowSOM analysis of monocytes confirmed manual gating with no significant differences in the number of classical or inflammatory monocytes. This is in contrast to Ritz et al (2011), who previously reported an increase in CD14^+^CD16^+^ inflammatory monocytes in CRPS, whereas there were 20% more CD14^+^CD16^+^ cells in our CRPS cohort, although this did not reach statistical significance. There was, however, a generalised increase in pAKT expression in all leukocytes, although it was strongest in myeloid cells (Fig. 4b). There was a selective increase in pMAPKAPK2 (Fig. 4d), pp38 (Fig. 4e), pPLC*γ*2 (Fig. 4f), pSTAT1 (Fig. 4g) and pSTAT5 (Fig. 4i) expression in myeloid cells, relative to lymphocytes, with phosphorylation of p38, STAT1 and STAT5 in particular indicative of pro-inflammatory activation.

#### Dendritic cells

**Fig. 5:**
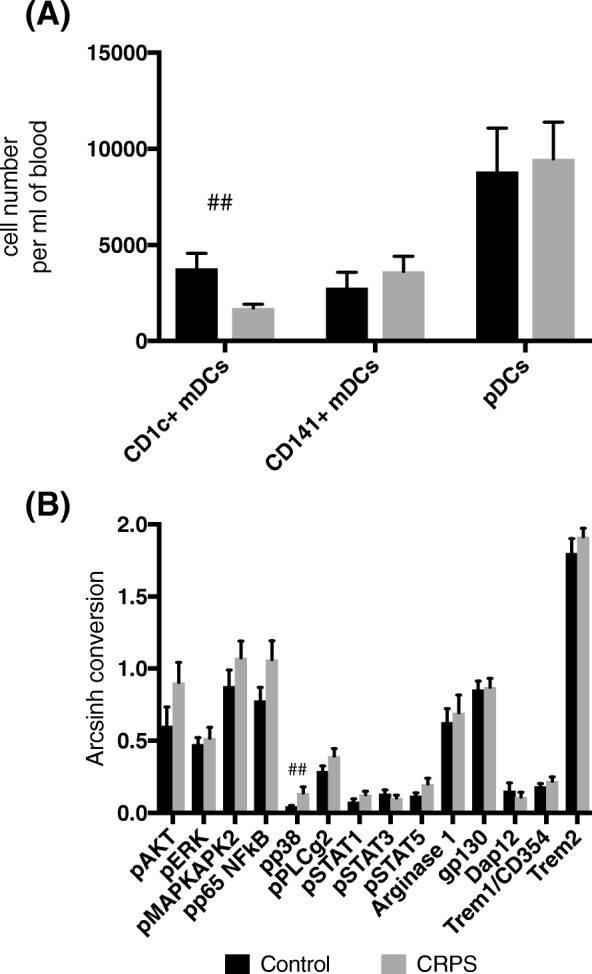
Reduced CD1c^+^ myeloid dendritic cell numbers but with increased p38 activation are found in the blood of CRPS participants compared to healthy controls. **a** A bar graph showing the number of cells in FlowSoM clusters representing the three major dendritic cell populations; CD1c^+^ mDCs (HLA-DR^+^CD14^−^CD11c^+^CD1c^+^CD141^−^), CD141^+^ mDCS (HLA-DR^+^CD14^−^CD11c^+^CD141^+^CD1c^−^) and plasmacytoid DCS (pDCs, HLA-DR^+^CD14^−^CD123^+^) in CRPS and control participants. **b** Intracellular signalling and functional marker expression levels in the FlowSoM cluster representing CD1c^+^ mDCs. *N* = 14 CRPS and *N* = 14 controls. All data are presented as group means (± S.E.M.). ^##^ represents *P* < 0.01 in a Mann-Whitney *U* Test

Unsupervised clustering using FlowSOM confirmed that there were significantly fewer CD1c^+^ mDCs (HLA-DR^+^CD14^−^CD11c^+^CD1c^+^CD141^−^) in CRPS (*U* = 34, *P* < 0.01), whilst the other two major dendritic cell populations, CD141^+^ mDCs and pDCs, were unchanged (Fig. 5a). Intracellular expression patterns within the CD1c^+^ mDC cluster of the CRPS group showed increased phospho-p38 expression compared to controls (*U* = 36, *P* < 0.01; Fig. 5b). There were no changes in intracellular expression in CD141^+^ DCs and pDCs (data not shown).

**Fig. 6:**
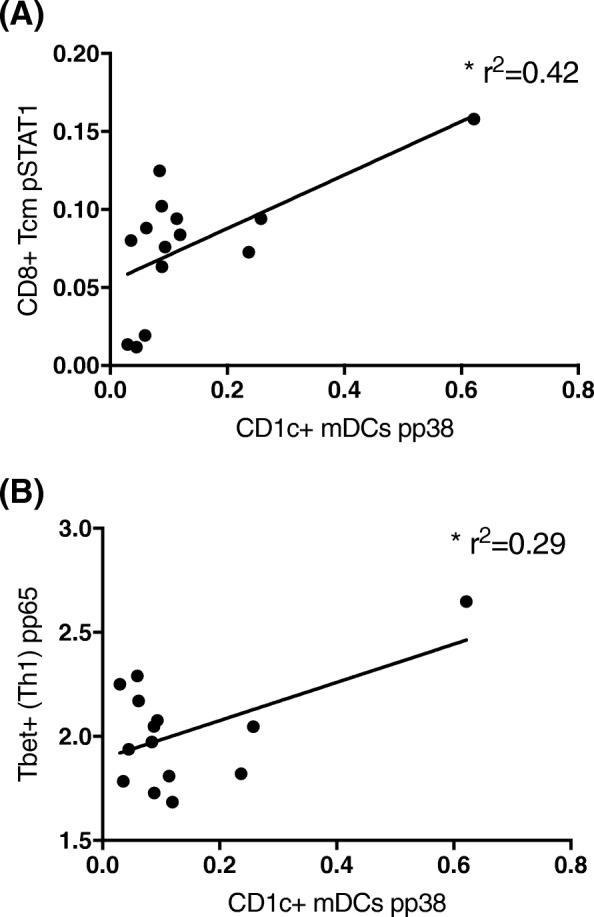
Increased pp38 signalling in CD1c^+^ myeloid dendritic cells correlates with increased signalling in T lymphocytes in the blood of CRPS participants. **a** Linear correlation between pp38 in CD1c + mDCs and pSTAT1 in central memory CD8^+^ T lymphocytes. **b** Linear correlation between pp38 in CD1c + mDCs and pP65 in central memory CD4^+^ Th1 lymphocytes. * represents *P* < 0.01 in a linear regression analysis

We probed the relationship of intracellular signalling in different cell types in individuals in the CRPS group and found that pp38 expression in CD1c^+^ mDC positively correlated with pSTAT1 expression in central memory CD8^+^ T lymphocytes (*F*_1,12_ = 8.52, *r*^2^ = 0.42, *P* < 0.05, Fig. 6a), and with pp65 expression in central memory Th1 lymphocytes (*F*_1,12_ = 4.98, *r*^2^ = 0.29; Fig. 6b). That is, activation of p38 in CD1c^+^ mDC is associated with increased activation markers in T lymphocytes.

## Discussion

Using a mass cytometric approach, we have identified increased numbers of central memory CD8^+^ and CD4^+^ T lymphocytes in 14 participants in the chronic phase of CRPS; moreover, these cells bare the hallmark of pro-inflammatory activation. Further, increased phospho-p38 expression provides evidence of CD1c^+^ mDCs activation, despite a reduction in their number. These findings provide preliminary evidence of an antigen-mediated T lymphocyte response in CRPS.

### CD8^+^ lymphocytes

Central memory CD8^+^ T lymphocytes show a selective expansion in CRPS, without changes to naïve and effector subsets. A previous study reported a reduction in total CD8^+^ T lymphocytes; however, they did not examine the central memory subtype and had a limited medication washout period [30]. Central memory CD8^+^ T lymphocytes persist in the blood, maintaining a strong proliferative capacity, but with reduced effector functions compared to other CD8^+^ T lymphocytes [46]. Central memory CD8^+^ T lymphocyte expansion is favoured following low-affinity antigen presentation via major histocompatibility complex (MHC) class I [47], although the exact antigen in CRPS remains unknown. It could be a damage-associated auto-antigen [48], or it may be related to an infection, given evidence of prior exposure to *campylobacter* in CRPS patients [49].

Central memory CD8^+^ T lymphocytes had increased expression of phosphorylated STAT1 and p65 (NF *κ* B subunit), as well as increased gp130 expression. NF*κ*B is a critical transcription factor in interferon-gamma (IFN*γ*) production in CD8^+^ T lymphocytes [50], whilst STAT1 signalling mediated by IFN*γ* regulates clonal expansion and production of memory CD8^+^ lymphocytes [51]. It is therefore possible that NF*κ*B-IFN *γ*-STAT1 signalling may be involved in the expansion of central memory T lymphocytes seen in CRPS. gp130 is a common signal transducing component of functional receptor complexes of the IL-6 family, including IL-6, IL-11, leukaemia inhibitory factor (LIF) and ciliary neurotrophic factor (CNTF) [52]. In CD8^+^ T lymphocytes, IL-6 binding to gp80 leads to the formation of a gp130 homodimer complex that results in activation of STAT3 and consequent effector functions mediated by IFN*γ* and granzyme B production [53–55]. CRPS patients have elevated IL-6 in blister fluid and CSF, as well as reduced plasma levels of soluble gp130, that counteracts IL-6 signalling [14, 17, 56, 57]. Therefore, it is possible increased gp130 expression by CD8^+^ lymphocytes may be related to increased IL-6 signalling resulting in effector functions that enhance cellular damage in CRPS. These cell signalling patterns indicate activation of the expanded central memory CD8^+^ T lymphocyte population and are consistent with their pathogenic role in CRPS.

### CD4^+^ Th1 lymphocytes

Central memory Th1 lymphocytes, but not other naïve or effector CD4^+^ T lymphocytes, show a selective expansion in CRPS. Relative to effector T lymphocytes, central memory CD4^+^ T cells are activated by lower levels of antigen presentation through MHC class II, have strong IL-2 mediated proliferative capacity but reduced production of effector cytokines, IL-4 or IFN-γ [58]. Under certain stimulation parameters, Th1 cells are much more likely to enter the memory pool than other helper T subtypes, such as Th17 [59]. In CRPS, the exact antigen that drives central memory Th1 lymphocyte expansion and consequently whether these cells are auto-reactive remains to be determined. Th1 responses are highly dependent on NF *κ* B signalling, being impaired by overexpression of the negative regulator, I*κ*B, as well as in the absence of p65 where IFN*γ* production was diminished [50, 60]. Thus, it is unsurprising to see that these cells have increased phosphorylation of p65 in CRPS. Increased tumour necrosis factor (TNF), IL-2 and soluble IL-2 receptor (sIL-2R) in the blood of CRPS patients provide supporting evidence of a Th1 response [19, 27]. An increase in central memory CD4^+^ T lymphocytes is also found in carpal tunnel syndrome [61], whilst in vivo adoptive transfer of Th1 cells in rats lacking mature T lymphocytes potentiated neuropathic pain symptoms [62]. Our findings, together with previous literature, support a role for central memory Th1 lymphocytes in the exaggerated nociceptive signalling associated with CRPS.

### CD4^+^ regulatory T lymphocytes

Central memory CD4^+^ Tregs are expanded and have increased phosphorylation of p65 and PLC*γ*2 in CRPS. Although NF*κ*B signalling has been shown to regulate development of Tregs [63], deletion of Ubc13, a positive regulator of I*κ*B, impairs their suppressive function and renders them sensitive to acquisition by Th1 cells [64]. Phosphorylated PLC*γ*2 is able to cleave phosphatidylinositol 4,5-bisphosphate (PIP2) into the second messengers phosphatidylinositol (3,4,5)-trisphosphate (PIP3) and diacylglycerol (DAG). DAG activates protein kinase C (PKC), which activates NF*κ*B signalling. Several PLC*γ*2 mutations have been linked to inflammatory and auto-immune diseases [65, 66].

A recent study that supports our findings reported an increase in CD39^+^ Tregs in CRPS, with concomitant decrease in Th17 cells, which they supress [29]. A similar imbalance of Th17 and Tregs was observed in chronic lower back pain and neuropathic pain [45, 67], although whether elevated Tregs contribute to pain or represent a compensatory response to persistent pain, stress and inflammation is unclear. Interestingly, in vivo expansion of Tregs has been shown to reduce symptoms of neuropathic pain, whilst Treg depletion increases pain hypersensitivity [68, 69]. Clearly, increased numbers of Tregs in CRPS patients is insufficient to suppress pain, which may be due to increased PLC*γ*2 and NF*κ*B signalling, which renders them sensitive to Th1 conversion.

### Dendritic cells

It is believed that in CRPS, on the production of damaged tissue, there is a release of substances such as damage-associated molecular patterns (DAMPS), which activate immature DCs in the skin and tissue [48, 70, 71]. This is supported by evidence of accumulation of dermal resident dendritic cells (i.e. Langerhans cells) in the affected limbs of CRPS patients with severe pain compared to the limbs of non-CRPS affected patients who had recovered from nerve injury, and in the tibial fracture mouse model of CRPS [21, 72]. These cells then become phenotypically mature and translocate from their resident position to the draining lymph node (axilla and groin) where they can activate CD8^+^ and CD4^+^ T lymphocytes [73–75].

We found a reduced number of circulating CD1^+^ mDCs, but with increased p38 phosphorylation. CD1c^+^ mDCs are strong stimulators of naive CD4^+^ T lymphocytes, and phosphorylation of p38 critically regulates the production of IL-12, a Th1 priming cytokine, in DCs [76]. Therefore, since increased CD1c^+^ mDCs p38 signalling correlated with increased NF*κ*B and STAT1 signalling in central memory CD4^+^ Th1 and CD8^+^ T lymphocytes respectively, we hypothesise that increased phospho-p38 expression in mDCs is related to T lymphocyte activation. Moreover, reduced numbers of circulating CD1^+^ mDCs could be indicative of trafficking to tissues or lymph nodes, given that reduced immature DCs have been reported in neurodegenerative diseases following brain trafficking [77]. Our findings together with those of others provide evidence of expansion of activated CD8^+^ and CD4^+^ T lymphocytes; activation of DCs, but with fewer found in the circulation, and larger numbers sequestrated in the affected peripheral tissue and draining lymph nodes [21, 72].

### Correlation of immune changes with pain and psychological variables

Activated CD4^+^ and CD8^+^ T lymphocytes contribute to nociceptive signalling and cellular damage through the release of cytokines or direct infiltration into peripheral nerves, dorsal root ganglia and the spinal cord [78, 79]. It is well known that cytokines and immune cells can signal to the brain through neural, humoral and cellular pathways and modulate mood [80, 81]. Although, it is yet to be confirmed whether T lymphocytes migrate to supraspinal sites in CRPS. Despite this, T lymphocytes can indirectly modulate pain processing in critical brain regions through peripheral inflammatory cascades [82–84].

The number of central memory CD8^+^ T lymphocytes positively correlated with stress scores, whilst the number of Th1 cells and their NF*κ*B signalling negatively correlated with anxiety scores and pain catastrophising, respectively. These results suggest that central memory CD8^+^ T lymphocyte expansion, rather than Th1 lymphocyte expansion and activation, may drive mood changes in CRPS. Elevated CD8^+^ T lymphocytes and decreased CD4^+^ T lymphocytes have been previously reported in human immunodeficiency virus (HIV) and liver cirrhosis patients with co-morbid depression [85–88]. Further, plasma levels of the archetypal pro-inflammatory cytokine TNF correlated with depression scores in painful neuropathy [89]. Therefore, our findings provide further evidence that peripheral and central inflammatory cascades may mediate the co-morbidity of pain and depression [44, 82, 84, 90].

### Limitations

We wish to emphasise that immune changes are only one aspect of the complex pathophysiology of CRPS and we have not addressed these multiple other areas of investigation such as neural, glial and cortical changes. We refer the reader to several current models that describe these changes in much detail [7, 9, 91]. The major limitations of this study are the small CRPS cohort, and despite being non-significant, the larger proportion of females in the CRPS group relative to the healthy control group. In particular, sex differences in immune function and pain processing could represent a confounding factor in our findings, given that in female mice T lymphocyte function appears to be more critical to the development of neuropathic pain than in male mice [92, 93]. Therefore, whilst expanded T lymphocyte populations in the CRPS group could be related to pain, one could also argue that such differences are exaggerated due to the female dominant make-up of the CRPS group, relative to the male dominant healthy control group.

It is also worth considering that our data demonstrate immune associations with CRPS, rather than causation. Immune changes could represent a pre-existing baseline due to infection or an auto-immune condition that is unmasked by trauma, or trauma-related markers rather than CRPS markers. Therefore, an injury control group where participants experienced a similar trauma but did not go onto develop CRPS would be important to demonstrate that immune changes are CRPS specific. Another factor worth considering is that the immune changes could be related to the stress which occurs due to living with a painful condition such as CRPS, as cortisol is known to modulate T lymphocyte polarisation [94]. This fact is further highlighted given the similar Treg profiles reported in other pain conditions [45, 67].

In light of these limitations, our findings should be considered preliminary and we recommend a large confirmatory study with balanced sexes and an injury control group be performed in the future. Such a study could also seek to immunophenotype patients across different phases of CRPS, and investigate the effects of medication to confirm if a 7-day wash-out period is significant.

## Conclusions

We have demonstrated a significant disruption to lymphocyte homeostasis in 14 participants in the chronic phase of CRPS. Expansion of central memory CD8^+^ and CD4^+^ T lymphocytes with distinct signalling activation patterns provides preliminary evidence of a chronic antigen-mediated T lymphocyte response in CRPS. Moreover, reduced numbers, but with increased p38 signalling in CD1^+^ mDCs, could implicate this dendritic cell type in T lymphocyte activation.

## Abbreviations

AKT: Protein kinase B
CD: Cluster of differentiation
CNTF: Ciliary neurotrophic factor
CRPS: Complex regional pain syndrome
DAG: Diacylglycerol
DAMPS: Damage-associated molecular patterns
DASS21: Short form depression, anxiety and stress scale
EDTA: Ethylenediaminetetraacetic acid
ERK: Extracellular signal-regulated kinase
FACS buffer: Fluorescence-activated cell sorting buffer
FCS: Fetal calf serum
.fcs: Flow cytometry standard
FoxP3: Forkhead box P3
gp130/CD130: Glycoprotein130
HLA-DR: Human leukocyte antigen-DR isotype
IFN*γ*: Interferon-gamma
IL: Interleukin
I*κ*B: Inhibitor of NF*k*B
LIF: Leukaemia inhibitory factor
M2: Muscarinic-2 acetylcholine receptor
MAPKAPK2: Mitogen-activated protein kinase-activated protein kinase 2
mDCs: Myeloid dendritic cells
MHC: Major histocompatibility complex
NF*k*B: Nuclear factor kappa-light-chain-enhancer of activated B cells
NSAIDs: Non-steroidal anti-inflammatory drugs
p38: p38 mitogen-activated protein kinase
p65: Subunit of NF*κ*B
PBMCs: Peripheral blood mononuclear cells
PBS: Phosphate-buffered saline
PCS: Pain catastrophizing scale
PE: Phycoerythrin
PFA: Paraformaldehyde
PIP2: Phosphatidylinositol 4,5-bisphosphate
PIP3: Phosphatidylinositol (3,4,5)-trisphosphate
PKC: Protein kinase C
PLC*γ*2: 1-Phosphatidylinositol-4,5-bisphosphate phosphodiesterase gamma-2
PSEQ: Pain self-efficacy questionnaire
S.E.M.: Standard error of the mean
SF-MPQ-2: Short form McGill pain questionnaire
sIL-2R: Soluble IL-2 receptor
SPADE: Spanning tree progression of density-normalised events
STAT: Signal transducer and activator of transcription
T-bet: T-box protein expressed in T lymphocytes
TCR*γδ*^+^: T lymphocyte receptor *γδ* ^+^ sub-unit
Th: T helper lymphocytes
TNF: Tumour necrosis factor
TREM: Triggering receptor expressed on myeloid cells
TSK: Tampa scale for kinesiophobia
VAS: Visual analogue scale
