# Correction to: Expansion and activation of distinct central memory T lymphocyte subsets in complex regional pain syndrome

**DOI:** 10.1186/s12974-019-1470-z

**Published:** 2019-04-03

**Authors:** Marc A. Russo, Nathan T. Fiore, Caryn van Vreden, Dominic Bailey, Danielle M. Santarelli, Helen M. McGuire, Barbara Fazekas de St Groth, Paul J. Austin

**Affiliations:** 1 Hunter Pain Clinic, 91 Chatham Street, Broadmeadow, NSW 2292 Australia; 2 Genesis Research Services, 220 Denison St, Broadmeadow, NSW 2292 Australia; 3 Discipline of Anatomy & Histology, School of Medical Sciences, Faculty of Medicine and Health, The University of Sydney, Room E513, Anderson Stuart Building, Sydney, NSW 2006 Australia; 4 Ramaciotti Centre for Human Systems Biology, Charles Perkins Centre, The University of Sydney, Sydney, NSW 2006 Australia; 5 Sydney Cytometry, Centenary Institute and the Charles Perkins Centre, John Hopkins Drive, Camperdown, NSW 2050 Australia; 6 Discipline of Pathology, School of Medical Sciences, Faculty of Medicine and Health, The University of Sydney, Sydney, NSW 2006 Australia

**Correction to: J Neuroinflammation**

**https://doi.org/10.1186/s12974-019-1449-9**

Following publication of the original article [1], the authors reported an error in Figure 4 as the wrong figure was used. In the incorrect Fig. 4, panels b-i were missing the numerical scale values. The original article has been corrected. The authors would like to apologize for this error.

**Fig. 4:**
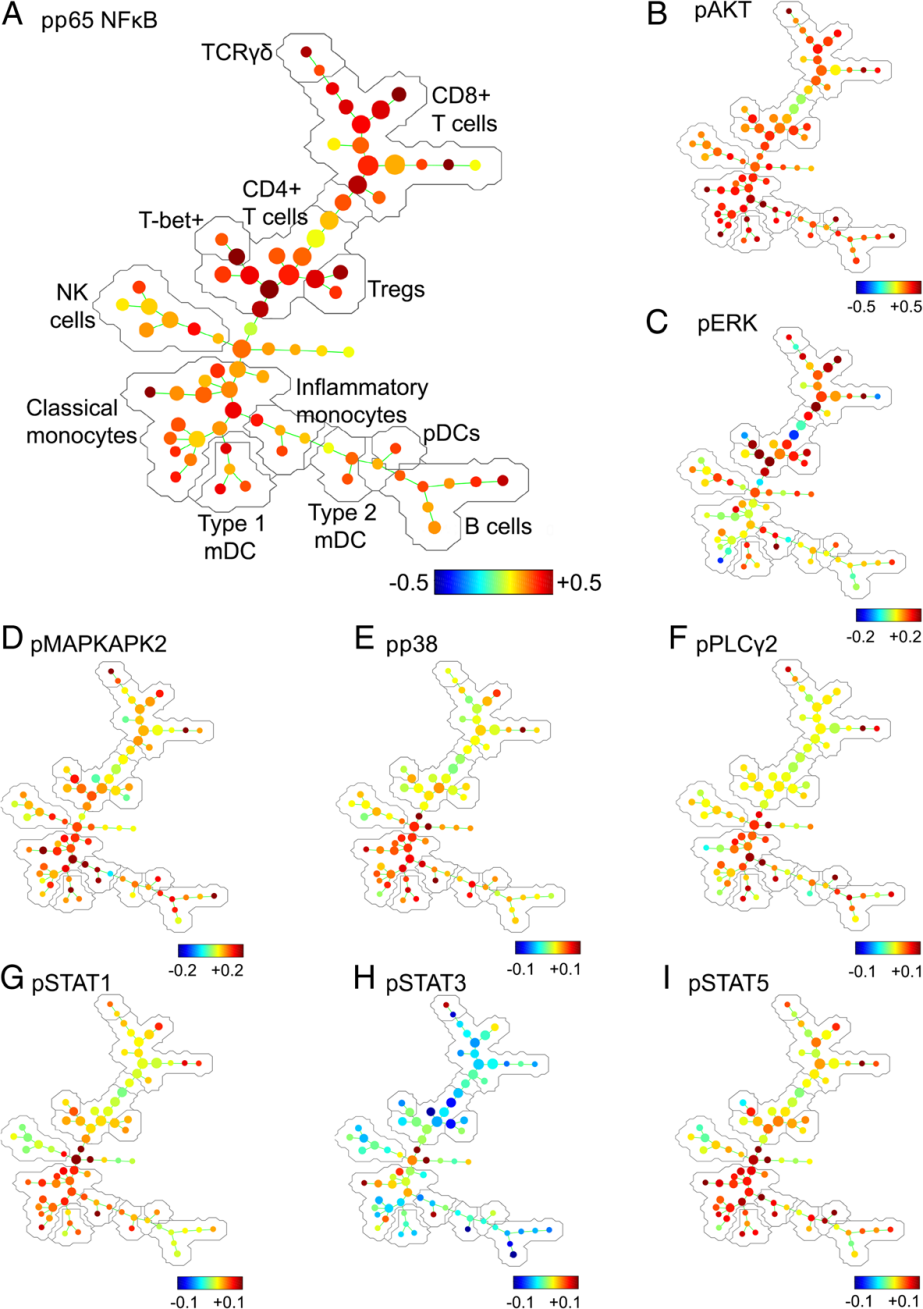
Spanning tree progression of density-normalised events (SPADE) trees showing evidence of pro-inflammatory activation in distinct lymphocyte and myeloid cell populations in the blood of CRPS participants relative to healthy controls. The SPADE algorithm was run on a downsampled population of single leukocytes from all CRPS and control participants. SPADE trees were generated showing the fold-change between CRPS and control groups in the expression of phosphorylated (activated), **a** p65 NF*κ*B, **b** AKT, **c** ERK, **d** MAPKAPK2, **e** p38, **f** PLC*γ*2, **g** STAT1, **h** STAT3 and **i** STAT5. The major cell populations labelled in (**a**) are representative of all SPADE trees. Note: colour scales vary for each marker
